# Transcranial magnetic stimulation with a half-sine wave pulse elicits direction-specific effects in human motor cortex

**DOI:** 10.1186/1471-2202-13-139

**Published:** 2012-11-05

**Authors:** Nikolai H Jung, Igor Delvendahl, Astrid Pechmann, Bernhard Gleich, Norbert Gattinger, Hartwig R Siebner, Volker Mall

**Affiliations:** 1 Department of Pediatrics, Technical University Munich, Kinderzentrum München gemeinnützige GmbH, Heiglhofstrasse 63, Munich, 81377, Germany; 2 Department of Paediatrics and Adolescent Medicine, Division of Neuropaediatrics and Muscular Disorders, University Medical Centre Freiburg, Mathildenstr 1, Freiburg, 79106, Germany; 3 European Neuroscience Institute Göttingen, Grisebachstr 5, Göttingen, 37077, Germany; 4 Central Institute for Medical Engineering at Technische Universität München (IMETUM), Boltzmannstrasse 11, Garching, 85748, Germany; 5 Danish Research Centre for Magnetic Resonance, Copenhagen University Hospital Hvidovre, Kettegaard Allé 30, Hvidovre, 2650, Denmark

**Keywords:** Transcranial magnetic stimulation, Current direction, Half-sine stimulus, I-waves, Stimulus–response curves, Motor cortex

## Abstract

**Background:**

Transcranial magnetic stimulation (TMS) commonly uses so-called monophasic pulses where the initial rapidly changing current flow is followed by a critically dampened return current. It has been shown that a monophasic TMS pulse preferentially excites different cortical circuits in the human motor hand area (M1-HAND), if the induced tissue current has a posterior-to-anterior (PA) or anterior-to-posterior (AP) direction. Here we tested whether similar direction-specific effects could be elicited in M1-HAND using TMS pulses with a half-sine wave configuration.

**Results:**

In 10 young participants, we applied half-sine pulses to the right M1-HAND which elicited PA or AP currents with respect to the orientation of the central sulcus.

Measurements of the motor evoked potential (MEP) revealed that PA half-sine stimulation resulted in lower resting motor threshold (RMT) than AP stimulation. When stimulus intensity (SI) was gradually increased as percentage of maximal stimulator output, the stimulus–response curve (SRC) of MEP amplitude showed a leftward shift for PA as opposed to AP half-sine stimulation. Further, MEP latencies were approximately 1 ms shorter for PA relative to AP half-sine stimulation across the entire SI range tested. When adjusting SI to the respective RMT of PA and AP stimulation, the direction-specific differences in MEP latencies persisted, while the gain function of MEP amplitudes was comparable for PA and AP stimulation.

**Conclusions:**

Using half-sine pulse configuration, single-pulse TMS elicits consistent direction-specific effects in M1-HAND that are similar to TMS with monophasic pulses. The longer MEP latency for AP half-sine stimulation suggests that PA and AP half-sine stimulation preferentially activates different sets of cortical neurons that are involved in the generation of different corticospinal descending volleys.

## Background

Transcranial magnetic stimulation (TMS) of the primary motor hand area (M1-HAND) can induce multiple descending corticospinal volleys which can be recorded from the upper spinal cord using invasive techniques [1,2]. These descending volleys are caused by either direct or indirect activation of fast-conducting pyramidal tract neurons that connect monosynaptically to spinal motoneurons [3]. According to their latency, these waves have consequently been termed direct waves (D-waves) and early or late indirect waves (I-waves) [4]. Early and late I-waves are thought to be generated transsynaptically by TMS-induced excitation of different intracortical circuits projecting onto the corticospinal neurons.

The recruitment pattern of these multiple descending volleys is not fixed but rather depends on the intensity of the TMS pulse and the direction of the current that is induced in M1-HAND [3]. If the coil is positioned over M1-HAND in a way that the main current in M1-HAND runs in a sagittal direction, TMS can be used to preferentially recruit different sets of I-waves. At stimulus intensities that are just suprathreshold for evoking a motor evoked potential (MEP), a single monophasic TMS pulse that induces a posterior-anterior (PA) current in the M1-HAND leads to preferential recruitment of early I-waves (I1) [5,6]. If the induced current has an anterior-posterior (AP) direction, later I-waves (I3) are primarily recruited [5,6]. Since early and late I-waves are thought to be generated by different intracortical circuits, it has been concluded that different sets of intracortical neurons are excited in the motor cortex when inverting the current direction of a monophasic stimulus from PA to AP or vice versa [5,7].

The pattern of evoked descending volleys also depends on the configuration of the TMS pulse [8]. An asymmetric “monophasic” pulse is mostly used when studying the physiology of human M1-HAND with single-pulse TMS, while a “biphasic” pulse configuration is commonly used for repetitive TMS [9]. The first phase of the “monophasic” pulse produces a strong initial current flow which lasts less than 100 μs, while the second phase produces a critically dampened return current lasting several 100 μs. Since only the first phase of the stimulus produces a current flow in the stimulated brain which is strong enough to elicit action potentials, monophasic single-pulse TMS are well-suited to investigate direction-specific effects of TMS on the excitation of corticospinal output neurons [7,10]. For biphasic TMS pulses – that were not investigated in the present study -, the direction of the current is reversed twice. Since the phase of the biphasic pulse is rapidly reversed, all current components induced by the different phases of the biphasic TMS pulse contribute to electrical cortex stimulation. In contrast to monophasic TMS, the tissue current induced by the second (reversal) phase is physiologically more effective than the current induced by the initial current phase when using a biphasic TMS pulse [11,12]. These differences between monophasic and biphasic TMS pulses also explain why different pulse configurations excite partly different sets of cortical axons when using the same coil orientation [8].

Like (asymmetric) monophasic pulses, a half-sine TMS pulse only induces a tissue current that flows in one direction without current reversal. Thus, single-pulse TMS using a half-sine pulse configuration should be suited to study direction-specific effects of TMS in the intact human M1-HAND. However, a previous TMS study failed to demonstrate any statistically significant direction-specific differences in MEP amplitude, latency or cortical motor threshold for AP versus PA currents when using a half-sine pulse configuration [13]. In the present study, we re-examined the question whether TMS pulses having a half-sine wave configuration can be used to examine direction-specific effects of TMS of the M1-HAND. Significant direction specific effects of half-sine pulses have not been shown so far and they might offer new opportunities to study direction specific effects of TMS and, prospectively, motivate to install plasticity inducing protocols that combine AP and PA oriented half-sine stimuli. This was possible by using a TMS device that was able to generate isolated negative and positive half-sine waves (P-Stim 160 stimulator, Mag & More GmbH, Munich, Germany). It was achieved by installing a second antiparallel connected thyristor (semiconductor switch) allowing the regulation of both polarities. Using either AP or PA stimulation with respect to the central sulcus, we assessed the direction-specific properties of the half-sine wave configuration on resting motor threshold (RMT), MEP amplitude and MEP latency without changing coil position. MEPs were recorded at rest from the left abductor pollicis brevis muscle (APB). Our basic assumption was that switching the current direction would result in a preferential activation of cortical circuits involved in the generation of early or late cortical I-waves. Hence, we expected RMT to be consistently lower, MEP amplitudes to be significantly higher, and MEP latencies to be consistently shorter for half-sine wave stimulation inducing a PA as opposed to an AP current in M1-HAND. The direction-specific effects on MEP amplitudes and latencies were assessed across a wide range of stimulus intensities which enabled us to construct stimulus–response curves (SRCs) for both, MEP amplitude and latency. SRCs (often referred to as input–output curve or recruitment curve) were obtained with and without adjustment for differences in RMT.

## Results

Participants tolerated the experiments well, reporting no adverse events. We found an identical “motor hotspot” for PA or AP half-sine stimulation.

### Experiment 1: Differences in RMT, MEP amplitude and MEP latency

**Figure 1:**
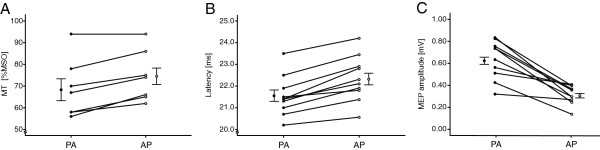
**Individual differences in resting motor threshold (RMT), mean MEP amplitude and mean MEP latency depending on the direction of the induced current in M1-HAND (Experiment 1, n = 10 for MEP and latency, n = 7 for RMT).** MEP were recorded from relaxed left APB muscle. MEPs were evoked with single-pulse TMS using a half-sine wave stimulus and a suprathreshold stimulus intensity to elicite mean MEP amplitude of 0.5 mV (SI_0.5mV_). (**A**) The resting motor threshold expressed in % of MSO was consistently lower for PA as opposed to AP stimulation. (**B**) Mean MEP latency of left APB was consistently shorter for PA as opposed to AP stimulation. (**C**) Mean MEP amplitudes of left APB were consistently larger for PA stimulation. PA: Half-sine wave stimulus inducing a posterior-anterior current in M1-HAND, AP: Half-sine wave stimulus inducing an anterior-posterior current in M1-HAND. Error bars indicate mean ± standard error of the mean.

We first assessed in this pilot experiment differences in RMT as well as differences in mean MEP amplitude and latencies for MEPs evoked at moderate suprathreshold intensity (Figure 1). Mean RMT was significantly lower when the half-sine stimulus produced a PA current as opposed to an AP current in the right M1-HAND (68.71 ± 5.17% for PA stimulation vs. 74.57 ± 4.45% for AP stimulation; *p *= 0.003). Apart from one participant with relatively high RMT above 90% MSO (94% MSO for AP and PA direction), the RMT values were always lower for PA half-sine stimulation, ranging from 56% to 94% of MSO. Mean MEP amplitudes were larger when the slightly suprathreshold stimulus induced a PA current direction in right M1-HAND (0.63 ± 0.17 for PA stimulation vs. 0.32 ± 0.08 mV for AP stimulation; *p *< 0.001). Mean MEP latencies were significantly shorter for the half-sine pulse inducing a PA directed current relative to TMS producing an AP directed current (21.54 ± 0.92 ms for PA stimulation vs. 22.34 ± 1.05 ms for AP stimulation; (*p *< 0.001).

### Experiment 2: SRC_MSO_ measurements for MEP amplitude and latency

**Figure 2:**
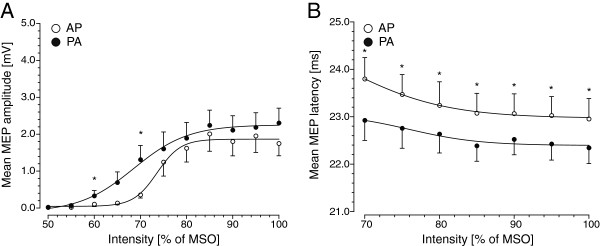
**Stimulus–response curves (SRCs) of the relaxed left APB muscle using a stimulus intensity referenced to maximal stimulator output.** Experiment 2; n=9). The SRCs reflect changes in mean MEP amplitude (panel A) or mean MEP latency (panel B) with increasing intensity of half-sine wave TMS. Twelve stimulus intensities were used, ranging from 50 to 100% of MSO. Filled circles refer to MEP evoked by a half-sine wave stimulus producing a PA current, whereas open circles refer to MEP evoked by a half-sine wave stimulus inducing an AP current in right M1-HAND. In six participants, MEP latencies were not reliably measurable at stimulus intensities between 50 to 65% of MSO. Therefore, changes in MEP latencies are only displayed from 70-100% of MSO upwards. Error bars represent standard error of the mean. % of MSO: percentage of maximum stimulator output. Asterisks indicate significant differences (paired *t*-test, Bonferroni corrected).

We examined whether the change in mean MEP amplitude and latency with increasing stimulus intensity depended on the direction of the induced current in right M1-HAND using stimulus intensities that were adjusted to maximal stimulator output (MSO). Figure 2 summarizes the group data. The SRCs of mean MEP amplitude had a sigmoid shape with a sigmoid increase in MEP amplitude at suprathreshold intensities which reached a plateau at very high stimulus intensities (Figure 2A). The SRCs of mean MEP latencies showed a gradual decrease with increasing stimulus intensity which was slightly more pronounced at lower stimulus intensities (Figure 2B). These patterns were consistently found for both PA and AP half-sine stimulation.

When stimulus intensities were referenced to MSO, flipping the current direction induced a significant shift of the SRC_MSO_. With respect to MEP amplitude, PA half-sine stimulation resulted in a notable leftward shift of the SRC_MSO_ compared to AP half-sine stimulation (Figure 2A). Boltzman function revealed V50 of 68.5 with a slope of 5.90 and a maximum of 2.25 mV for PA and a V50 of 73.61 with a slope of 2.55 and a maximum of 1.87 mV for AP stimulation. The SRC_MSO_ obtained with PA half-sine stimuli started to rise earlier than the SRC_MSO_ obtained with AP half-sine stimuli, while the maximal MEP amplitude did not differ (Figure 2A). Accordingly, rmANOVA showed significant main effects of CURRENT DIRECTION (*F*_[1;16]_ = 11.48, *p *= 0.010) and INTENSITY (*F*_[14;252]_ = 22.08, *p *< 0.001), as well as for the interaction of both CURRENT DIRECTION*INTENSITY (*F*_[14;252]_ = 2.30, *p *= 0.008).

The SRC_MSO_ for mean MEP latency also showed a direction-specific shift (Figure 2B). When an AP half-sine stimulus was used, SRC_MSO_ displayed an upward shift of the curve relative to PA stimulation due to an increase of approximately 1 ms in MEP latency across the whole intensity range. This was confirmed by the rmANOVA which yielded a significant effect of CURRENT DIRECTION (*F*_[1;16]_ = 59.91, *p *< 0.001) and INTENSITY (*F*_[6;108]_ = 10.0, *p *< 0.001), but no significant interaction CURRENT DIRECTION*INTENSITY (*F*_[6;108]_ = 0.74, *p *= 0.620).

### Experiment 3: SRC_RMT_ measurements for MEP amplitude and latency

**Figure 3:**
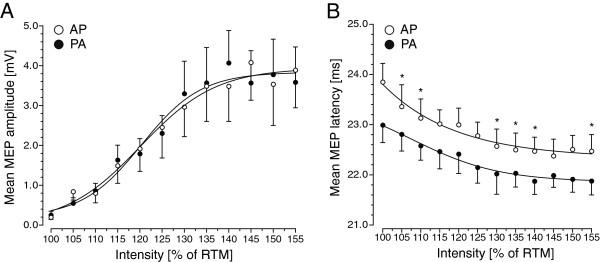
**Stimulus–response curves (SRCs) of the relaxed left APB muscle using a stimulus intensity referenced to individual resting motor threshold (RMT) for AP and PA half-sine stimulation (Experiment 3, n = 8).** SRCs of the relaxed left APB muscle in RMT indicate changes in mean MEP amplitude (panel A) or mean MEP latency (panel B) with increasing intensity of half-sine wave TMS. For the SRCs, the increase in stimulus intensity was individually adjusted to the respective RMT intensity for AP and PA stimulation (SRC_RMT_) to control for direction-specific differences in RMT. Filled circles refer to the mean MEP amplitude or latency evoked by a half-sine wave stimulus producing a PA current, whereas open circles refer to the mean MEP amplitude or latency evoked by a half-sine wave stimulus inducing an AP current in right M1-HAND. Error bars represent standard error of the mean. Asterisks indicate significant differences (paired *t*-test, Bonferroni corrected).

Since the current direction had a consistent influence on RMT (experiment 1), we also assessed the change in mean MEP amplitude and latency with increasing stimulus intensity taking into account the differences in RMT. Stimulus intensities were calibrated to the individual RMT as obtained with PA or AP stimulation (SRC_RMT_). RMTs ranged from 51% MSO to 62% MSO (mean: 56.1%± 3.60 MSO) for PA and from 60% MSO to 65% MSO (mean: 63.1%± 2.4 MSO) for AP current direction. Hence, stimulation intensities ranged from 51% MSO to 96% MSO for PA and from 60% MSO to 100% for AP. Group data are illustrated in Figure 3. When adjusting the stimulus intensity to the individual RMT intensity for PA and AP stimulation, the superimposed SRC_RMT_ of mean MEP amplitudes showed no differences in amplitude gain with increasing intensity between both current directions (Figure 3A). Boltzman function revealed V50 of 120.37 with a slope of 6.71 and a maximum of 3.84 mV for PA and a V50 of 120.14 with a slope of 8.52 and a maximum of 3.94 mV for AP stimulation. This was confirmed by the rmANOVA. While there was a main effect of INTENSITY (*F*_[11;154]_ = 12.12, *p *< 0.001), rmANOVA revealed no main effect of CURRENT DIRECTION (*F*_[1;14]_ = 0.002, *p *= 0.970) and no interaction between the two factors CURRENT DIRECTION*INTENSITY (*F*_[11;154]_ = 0.60, *p *= 0.820).

In contrast, the difference in mean MEP latency was still present when SRCs were adjusted for differences in RMT between AP and PA half-sine stimulation. For mean MEP latency, the SRC_RMT_ still showed a significant upward shift for AP as opposed to PA half-sine stimulation due to consistently longer MEP latencies for AP stimulation across the entire range of intensities (Figure 3B). Accordingly, the rmANOVA showed a main effect of INTENSITY (*F*_[11;154]_ = 15.33; *p *< 0.001) and CURRENT DIRECTION (*F*_[1;14]_ = 66.15, *p *< 0.001) without a significant interaction between the factors CURRENT DIRECTION*INTENSITY (*F*_[11;154]_ = 1.18, *p *= 0.845).

## Discussion

Using the MEP as a measure for TMS-induced excitation of the fast-conducting corticospinal neurons, our results provide converging evidence for direction-dependent effects of half-sine TMS over the M1-HAND. TMS was associated with significantly lower RMT when the half-sine pulse induced a PA current in the right M1-HAND as opposed to an AP current. Half-sine stimulation causing a PA current in M1-HAND also resulted in higher MEP amplitudes and shorter MEP latencies than AP half-sine stimulation. These results significantly extend a previous study [13] in which no consistent differences in RMT, MEP latency or amplitude were found between AP and PA stimulation when single-pulse TMS used a half-sine pulse configuration.

### Direction-specific effects on cortical motor threshold

The cortical motor threshold is a well-established measure of overall corticospinal excitability and commonly used to individually adjust the intensity of TMS [9]. Since the cortical motor threshold can be increased by administration of drugs that block voltage-gated sodium channels [14], it reflects how efficiently the TMS-induced tissue current polarizes cortical neurons and, hereby, transsynaptically activates corticospinal output neurons. In this study, all participants showed lower RMT when half-sine stimulation induced a PA current in the M1-HAND, apart from one participant with a very high RMT (94% MSO for AP and PA direction).

This finding extends previous studies which have consistently shown a direction-dependent effect on RMT for monophasic and biphasic pulse configurations [11,13]. For monophasic pulse configurations, it has been shown that a current passing the M1-HAND in a PA direction perpendicular to the central sulcus is more effective than one in the opposite direction [11,15,16]. For the biphasic pulse waveform, the most effective current direction to evoke MEPs in M1-HAND is opposite to that for the monophasic pulse. We found that less current is needed to evoke a MEP when the first phase of the half-sine stimulation induced a PA current in M1-HAND. This indicates a marked asymmetry between the first and the second phase of the half-sine stimulus in terms of their respective contributions to transsynaptic corticospinal excitation. Further, in analogy to a monophasic pulses, our RMT results imply that it is the first phase of the half-sine pulse that makes the strongest contribution to the excitation of cortical neurons. In contrast to biphasic pulses, the second reversal phase of the half-sine pulse does not play a predominant role, presumably because of its relatively shorter duration and smaller amplitude. Cortical axons have a wide range of orientations relative to the applied electric field, due to different orientations within the cortex and to cortical folding [17]. Our RMT measurements are in good agreement with the predictions made by recent modelling work to determine the preferential stimulation site of TMS-induced neural excitation. Using the finite element method, it was concluded that cortical neurons were most easily stimulated by a PA directed electric fields (except association fibers) using mono- and biphasic waveforms [17].

In contrast to our results, the orientation-specific difference in RMT did not reach significance for half-sine pulse stimulation in a previous study [13]. Several methodological differences might account for this discrepancy: The study by Sommer et al. [13] stimulated the left M1-HAND and used a MagPro X100 MagOption stimulator and a slightly bent figure-of-eight coil with overlapping windings in the coil centre (Magventure, Skovlunde, Denmark). In addition, they used the relative frequency method based on the Rossini-Rothwell criterion [18] to estimate RMT which might have been less precise as the adaptive method employed in the present study method [9].

### Direction-specific effects on the intensity-dependent gain in MEP amplitude

The SRC describes the increase in MEP amplitude with increasing TMS intensity [19]. The SRC usually has a sigmoid shape with a horizontal part below motor threshold, a sigmoid slope at suprathreshold intensities that reaches a horizontal plateau at very high stimulus intensities. While the cortical motor threshold reflects the excitability of a core of cortical neurons that are most excitable, the SRC measure reflects the efficacy of TMS to additionally excite cortical neurons that are intrinsically less excitable or spatially further from the “hot spot” of neural excitation [20]. When using fixed stimulus intensities that were referenced to MSO and thus ignored inter-individual differences in individual RMT, the resulting SRC_MSO_ showed a leftward shift of the SRC for PA as opposed to AP half-sine stimulation. One possible explanation of this result may be the fact that with PA stimulation is more effective (resulting in lower RMTs) and preferentially I1-waves are excited and this in turn leads to a larger accumulation of descending corticospinal volleys. The direction-specific differences in SRCs were no longer present when the SRC curves were referenced to individual RMT. Indeed, the SRC_RMT_ for PA and AP half-sine pulse configuration were closely overlapping, showing a comparable gain function reaching the plateau at a stimulus intensity of approximately 140% of RMT. This is in agreement with the results of Sommer et al. [13] who found no differences in the initial slope of the SRC for PA and AP half-sine stimulation. The “jump” of the slope of SRCs in experiment 2A might contribute to the fact that 70% MSO was slightly suprathreshold for PA stimulation but around RMT for AP stimulation whereas 65% MSO was mainly subthreshold and 75% mainly suprathreshold for PA and AP stimulation. Together, the data suggest that the differences in the gain function as reflected by the SRC are mainly related to differences in the RMT, but that otherwise the quantitative recruitment of corticospinal output neurons with increasing stimulus intensities is comparable for PA and AP half-sine pulse configuration.

### Direction-specific effects on corticomotor latency

Another novel finding was the demonstration of a consistent difference between PA and AP half-sine stimulation regarding mean MEP latency. When TMS induced a PA directed current in the M1-HAND, MEP latency was approximately 1 ms shorter than with TMS inducing an AP directed current. One possible explanation is that, depending on the current direction, the half-sine stimulus excited different sets of cortical neurons that are presynaptic to the corticospinal output neurons and are involved in the generation of different I-waves. It is conceivable that the PA half-sine pulse, as it has been shown for the PA monophasic pulse [5], excited neural circuits involved in the generation of the I1-wave. This notion is also supported by the observation that the MEP latencies induced by a PA monophasic and PA half-sine pulse are very similar [13]. If this is the case, the latency difference between PA and AP half-sine stimulation of approximately 1 ms suggests that the AP half-sine pulse preferentially excited neural circuits contributing to the I2-wave.

Interestingly, this difference in MEP latency did not diminish with increasing stimulus intensity. Both, SRC_MSO_ and SRC_RMT_ measurements yielded a “latency jump” of approximately 1 ms that was stable across the entire intensity range. This finding indicates that AP half-sine stimulation consistently activates neural circuits that are more remote from the corticospinal output neurons than PA half-sine stimulation. Since this difference was not blurred at high stimulus intensities, we hypothesize that AP and PA stimulation might target distinct cortical patches in the M1-HAND. Indeed, it has been claimed that two different networks are stimulated by PA and AP stimulation [21]. Our results motivate for the use of paired TMS protocols which combine PA and AP stimulation with half-sine waveforms to study the intracortical interaction between these networks.

Beside the aforementioned neurophysiological parameters of TMS, current direction is also relevant for plasticity inducing protocols such as paired associative stimulation [22] or repetitive TMS [23] as it has been demonstrated for monophasic waveforms. Kujirai and co-workers concluded that the observed effects are due to the fact that AP-oriented monophasic TMS more readily activates I3 inputs to corticospinal neurons and that these in turn are an important component of associative plasticity in M1 [22]. Adjacent to studies investigating motor cortex plasticity, an influence of current direction on paired pulse protocols [24] and on intracortical inhibition (cortical silent period) has been demonstrated [25-27]. These results confirm the cortical origin of the different properties of PA and AP current direction in the brain. The notion that AP and PA oriented monophasic stimuli excite different intracortical neuronal circuits has recently motivated to use an intracortical paired association stimulation (PAS) protocol that combines AP and PA oriented monophasic stimulus pairs to induce spike-timing dependent-like plasticity in human M1-HAND [28]. The same concept might also motivate the use of an intracortical PAS protocol that combines AP and PA oriented half-sine stimuli.

## Conclusions

Using half-sine pulse configuration, single-pulse TMS elicits consistent direction-specific effects in M1-HAND that are similar to TMS with monophasic pulses. The longer MEP latency for AP half-sine stimulation suggests that PA and AP half-sine stimulation preferentially activates different sets of cortical neurons that are involved in the generation of different corticospinal descending volleys.

## Methods

### Participants and study design

All participants were self-reported right-handed and none had a history of neurological illness or fulfilled any other exclusion criteria concerning the safety of TMS [29]. The study was approved by the local ethics committee of the university hospital of Freiburg, Germany and was conducted according to the latest version of the Declaration of Helsinki. After full disclosure of the purpose and risks of the study procedure, all participants gave their written informed consent.

### Transcranial magnetic stimulation

Single half-sine pulses were applied to the right M1-HAND at a frequency of 0.1 Hz. TMS was performed with a P-Stim 160 Stimulator (MAG & More GmbH, Munich, Germany) with a pulse duration of 80 μs connected to a figure-of-eight shaped stimulation coil. Each wing of the coil had an outer diameter of 100 mm. The polarity of the half-sine pulse could be reversed, hereby flipping the direction of the induced current in the M1-HAND. We refer to PA stimulation when the initially induced current in right M1-HAND had a posterior-to-anterior direction. Conversely, AP stimulation refers to half-sine stimulation producing an anterior-to-posterior current flow in the right M1-HAND.

The figure-of-eight shaped coil was placed tangentially on the participant’s head with the centre of the coil overlying the M1-HAND of the non-dominant right hemisphere. We used the non-dominant hemisphere to avoid unspecific training effects of the dominant hemisphere and to stimulate as naïve as possible cortical areas. The handle of the coil pointed from posterolaterally to anteromedially at an angle of approximately 45° with respect to the midsagittal line. At this orientation, the induced tissue current in M1-HAND is directed roughly perpendicular to the central sulcus and is therefore optimal for activating the neurons of the corticospinal pathways transsynaptically [30].

We first identified the optimal position where a clearly suprathreshold half-sine stimulus elicited MEP of maximal amplitudes in the target muscle using AP and PA current orientation. By moving the coil over the M1-HAND while administering stimuli of suprathreshold intensity, we identified the optimal position for eliciting MEP of maximal amplitudes from the target muscle (“motor hotspot”) and marked this point with a felt tip pen on the scalp. The RMT of the left APB was determined using an adaptive method [9]. Specifically, we used a maximum-likelihood algorithm [31] and based the estimation of RMT on a set of 16 consecutive stimuli applying a cut-off value for peak-to-peak MEP amplitude of 0.05 mV [32].

### Experiment 1

Ten participants (mean age 24.8 ± 1.81 years, 7 females, 3 males) participated in experiment 1 in which we assessed the RMT as well as mean MEP amplitude and latency at a fixed stimulus intensity using a PA or AP half-sine pulse. Three participants did not take part in RMT measurements for private reasons. We used the following procedure to determine the stimulus intensity for eliciting MEPs: stimulus intensity was set at a fixed suprathreshold intensity which corresponded to the stimulus intensity at which a combination of alternating PA and AP half-sine stimuli elicited MEP with mean amplitude of 0.5 mV in the fully relaxed APB (SI_0.5mV_). 10 MEP were recorded for each current direction with the order of AP and PA stimulation being counterbalanced across participants.

### Experiment 2 and 3: Stimulus-response curves

Experiment 2 and 3 tested the gradual change in MEP amplitude and latency with increasing stimulus intensity for both AP and PA half-sine stimulation. In one out of the 10 participants we could not assess the SRC because of a high RMT. Experiment 2 and 3 only differed in the way the stimulus intensity was determined for assessing the stimulus–response curve (SRC). Ten MEP were recorded for each intensity level. To avoid hysteresis effects in recording IO curves, both current direction and intensity were delivered in a randomized order [33]. The sigmoidal Boltzman function was used to fit these data given by the equation:

$$Y=Max+\frac{Min-Max}{1+\exp\left(\frac{X-V50}{slope}\right)}$$

where *X* is SI, *Min* and *Max* are the minimum and maximum and *V50* is the SI halfway between *Min* and *Max. Slope* is a measure of the steepness of the curve.

In experiment 2, the TMS intensity was referenced to the percentage of MSO and the same stimulus intensities were set in all participants to assess the SRC (referred to as SRC_MSO_). The stimulator output was varied between 50% and 100% MSO in 5% MSO steps resulting in 11 stimulus intensity levels. Nine participants (26.4 ± 2.24 years, 4 females, 5 males) took part in experiment 2 (of whom *n *= 2 took also part in experiment 1 and 3).

In experiment 3, stimulus intensity was individually adjusted to participant’s RMT (referred to as SRC_RMT_). RMT was determined separately for half-sine stimuli producing AP or PA stimulation. 12 intensity levels were used which ranged from 100% to 155% of RMT separated by steps of 5% of RMT. Hence, the relative increase in stimulus intensity per intensity level differed among participants according to their RMT. Eight participants (26.38 ± 1.85 years, 4 females, 4 males) took part in experiment 3, of whom *n *= 3 also took part in experiment 1 and *n *= 2 in experiment 2.

### Electromyographic recordings

Participants were seated comfortably in an armchair during the experiments. Both arms were supported by a pillow. MEPs were recorded from the left APB using Ag-AgCl electrodes (AMBU, Ballerup, Denmark, surface area: 263 mm^2^) mounted in belly-tendon technique. Participants were asked to relax the target muscle during all measurements. Data were band-pass filtered (20 – 2000 Hz) and amplified using an Ekida DC universal amplifier (EKIDA GmbH, Helmstadt, Germany), digitized at a sampling rate of 5 kHz (MICRO1401*mk*II*,* Cambridge Electronic Design Ltd, Cambridge, UK) and stored on a standard personal computer for online visual display and later offline analysis using Signal software (Version 3; Cambridge Electronic Design Ltd, Cambridge, UK).

### Data analysis

Relaxation of the target muscle was monitored online using visual feedback of the electromyographic activity recorded from the APB. Additionally, each MEP sweep was inspected offline for the presence of voluntary muscleactivity. If the MEP sweep showed electromyographic activity with a peak-to-peak amplitude of >0.05 mV, the trial was excluded from analyses. Mean MEP amplitude was determined by measuring the two highest peaks of opposite polarity [34] and then averaged over 10 trials for each point of investigation (i.e. stimulus intensity). Latency was measured from stimulus artefact to the onset of MEP for each trial and then averaged.

Statistical analysis was performed using software (SPSS version 15.0, SPSS Inc., Chicago, IL, USA). Separate parametric *t*-tests for paired samples were computed to assess direction-specific differences in RMT as well as mean MEP amplitudes and latencies obtained in experiment 1.

The IOC_RMT_ and IOC_MSO_ were statistically analysed using repeated measures analysis of variance (rmANOVA) with the within-subject factors CURRENT DIRECTION (2 levels, AP versus PA current direction) and INTENSITY (12 levels) after the Kolmogorov-Smirnov test had revealed no violations of the assumption of normality. If necessary, Greenhouse-Geisser corrections were applied to account for violations of the assumption of sphericity, resulting in adjusted p-values based on adjusted degrees of freedom. For all statistical tests, significance level was set at 0.05. Bonferroni correction was applied to control for an increased risk for α-errors caused by multiple non-independent comparisons. All values are given as mean ± standard deviation, if not indicated otherwise.

## Competing interests

The authors declare that they have no competing interests.

## Authors’ contributions

NHJ drafted the manuscript, had substantial contributions to the research design as well as to acquisition, analysis and interpretation of data. ID had substantial contributions to research design, analysis and interpretation of data. AP had substantial contributions to the acquisition and analysis of data. BG and NG had substantial contributions to research design. HRS had substantial contributions to analysis and interpretation of data and VM had substantial contributions to research design, acquisition, analysis and interpretation of data. All authors read and approved the final manuscript and had complete access to the study data that support the publication.
